# Optimization of All-Polymer Optical Fiber Oxygen Sensors with Antenna Dyes and Improved Solvent Selection Using Hansen Solubility Parameters

**DOI:** 10.3390/s21010005

**Published:** 2020-12-22

**Authors:** Rune Inglev, Emil Møller, Jonas Højgaard, Ole Bang, Jakob Janting

**Affiliations:** 1 Department of Photonics, Danish Technical University, Ørsteds Plads bldg. 343, 2800 Kongens Lyngby, Denmark; ruing@fotonik.dtu.dk (R.I.); oban@fotonik.dtu.dk (O.B.); 2 Pisco Group, Rønnegade 1, 3. th., 2100 Copenhagen Ø, Denmark; emoller@piscogroup.dk (E.M.); jhojgaard@piscogroup.dk (J.H.)

**Keywords:** polymer optical fibers (POF), photoluminescence, Hansen Solubility Parameters (HSP)

## Abstract

We present an all-polymer optical fiber sensor for the sensing of dissolved oxygen by phase-fluorometry. The sensing matrix is applied as a film on the fiber end-surface, and consists of poly-methylmethacrylate (PMMA), the oxygen quenchable luminophore platinum-octaethylporphyrin (PtOEP) and the luminophore coumarin 545T for increasing the brightness of PtOEP by way of resonance energy transfer (RET), also called light harvesting. We show that by using Hansen Solubility Parameters (HSPs), it is possible to quantitatively formulate a solvent mixture with a good solubility of the polymer matrix and the luminophores simultaneously. Our approach can readily be extended to other polymers and luminophores and is therefore a valuable tool for researchers working with photoluminescence and polymeric matrices.

## 1. Introduction

Polymer Optical Fiber (POF) sensors with their distal end functionalized for sensing have been around since the early days of fiber-optical sensing. In 1980 Peterson et al. [1] demonstrated the first fiber optical pH probe, based on the principle of having a chamber with an indicator-dye bonded to the end of a pair of fibers. The same principle was soon thereafter used to demonstrate the first fiber-optical oxygen sensor [2]. Incidentally, this is also the first fiber-optical oxygen sensor based on a POF. Most of the development in fiber-optical oxygen sensing since then has been done with glass optical fibers.

In 1988 Lippitsch et al. [3] presented the first fiber-optic oxygen sensor based on the principle of phase-fluorometry. The measurement was performed using modulated excitation light at 460 nm and measuring the phase delay of the 610 nm emission from a ruthenium complex. This was not done with a POF, but the principle of fiber-optical phase-fluorometry began here, and in our work we will also utilize this technique.

The 1990s and 2000s saw a few contributions to the field of POF oxygen sensing. Notably by Morisawa et al. [4], Toba et al. [5], Chu and Lo [6] and Chu et al. [7]. Of these, only Morisawa et al. used a polymer matrix coated onto the fiber. Toba instead doped a poly-methylmethacrylate (PMMA) POF by immersing it in a solution of dichloromethane, ethanol and the luminophore Solvent Green 5, which effectively resulted in the luminophore being in a polymeric matrix inside the core.

In the 2010s Pulido and Esteban did work on tapering POFs for increased photoluminescence in a side-illuminated oxygen sensor [8,9]. Chen et al. investigated the effect different polymer matrices have on the characteristics of the sensor [10,11], while Fischer and Koop-Jakobsen developed a multi-fiber optode (MuFO) by combining many POFs together in a sensing array [12]. In their work, planar thin film sensing spots were attached onto the end of the POFs. The spots were made separately on a Polyethylene terephthalate (PET) foil by coating it with a thin layer of polystyrene and luminophore solution. Other contributions to POF oxygen sensing in the 2010s have mainly been using sol-gels as matrices for the luminophores [13,14,15,16].

**Figure 1:**
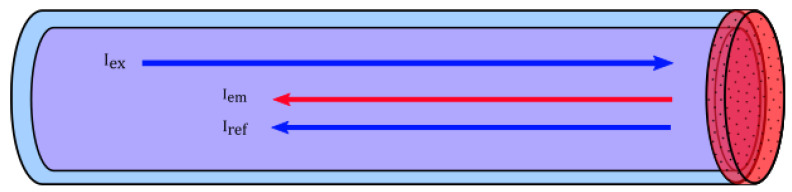
Concept for the fiber-optical sensor. The end-surface of the fiber is coated with a thin layer of polymer containing the oxygen-sensitive platinum-octaethylporphyrin (PtOEP). Excitation light will travel through the fiber and interact with the sensing matrix. Photoluminescence emission and reflected excitation light will return through the fiber, and can be separated by suitable filtering.

We present a POF sensor for dissolved oxygen using phase-fluorometry, in which the sensing matrix at the distal end of the fiber is of the same polymeric material (PMMA) as the fiber core (see Figure 1). When the sensing gel/matrix is applied to the fiber end, the solvent in the gel will partly dissolve the core material, and the two phases (sensing matrix and core) will entangle to provide a strong adhesion between the matrix and fiber. This will result in an increased robustness of the sensor compared to a situation in which the materials are different.

In addition, we show how Hansen Solubility Parameters (HSPs), can be used to engineer solvent mixtures that optimize the solubility of both polymer and luminophores. We also show how the solubility of the luminophore in the solvent is an important factor for the intensity of the photoluminescent emission from the sensing point. This approach can be readily extended to other polymers and luminophores, and is therefore of great use to researchers working with photoluminescence and polymeric matrices.

### 1.1. Hansen Solubility Parameters

The Hansen Solubility Parameters (HSPs) describe the cohesive energy density, $\delta^{2}$, of the interaction between molecules by a set of three parameters [17,18].

(1) $$\delta^{2}=\delta_{D}^{2}+\delta_{P}^{2}+\delta_{H}^{2}$$

The cohesive energy density is given by the secondary bonding forces (cohesive forces), when considering a unimolecular mix. The energy can be thought of as the energy it takes to remove a unit volume of molecules from the mix, divided by the volume removed, and it is a result of the dispersive, polar and hydrogen bonding between the molecules. The dispersive forces are quantified by $\delta_{D}$, the polar forces by $\delta_{P}$ and the hydrogen-bonding forces by $\delta_{H}$. Solvents and polymers can all be described by sets of these parameters and can therefore be thought of as points in a 3-dimensional space. In order to determine the solubility of a polymer in a given solvent, the “distance”, *R*, between the two must first be calculated. This is done by way of the following formula.

(2) $$R=\sqrt{4{\left(\delta_{D1}-\delta_{D2}\right)}^{2}+{\left(\delta_{P1}-\delta_{P2}\right)}^{2}+{\left(\delta_{H1}-\delta_{H2}\right)}^{2}}$$

The factor of 4 in-front of the dispersive energy density contribution is attributed to the fact that, contrary to the hydrogen and polar bonding forces, the dispersive forces do not care about direction. The factor can thus be thought of as a steric factor [18]. The distance measured is then compared to an “interaction distance”, $R_{0}$, for the polymer. If $R<R_{0}$, then the polymer is soluble in the particular solvent. If $R>R_{0}$ the polymer is not soluble. At $R=R_{0}$, the solubility will likely depend on other factors, such as entropy [19] or a more detailed description of the internal forces. The solubility can be graphically depicted in a 3-dimensional “HSP-space”, with the three energy densities corresponding to the three coordinates. The polymer HSPs and its interaction distance describes an ellipsoid in this space, and solvents will occupy points in the space. It is customary to either use $2\delta_{D}$ as the coordinate along one axis, or to shrink the axis for $\delta_{D}$, such that the ellipsoid looks like a sphere. Therefore, in Hansen Solubility Theory one often talks about the “solubility sphere”, even though it is an ellipsoid. The HSPs and interaction radii for polymers are found experimentally by testing with different solvents, with known HSPs, and labelling them as either “good” or “bad” depending on whether they dissolve the polymer. The HSPs and radii are then found by numerically fitting the data to the equation for *R* and the knowledge that “good” solvents should have $R<R_{0}$.

The HSPs for mixtures of solvents are easily computed, as they are simply the volumetric average of the HSPs for the individual solvents. In earlier work, we have used Hansen Solubility Parameters to investigate the etching/tapering of POFs made of different polymers, such as PMMA, Zeonex and TOPAS [20].

### 1.2. Extending HSPs to Photoluminescent Compounds

Hansen Solubility Parameters were developed for the paint and coatings industry, but there have been several authors who have investigated the use of HSPs for particles and pigments alike [17,21,22,23].

The determination of HSPs for particles can be done in several ways. Süß et al. presented a routine based on analytical centrifugation [23]. However, we have instead used an approach similar to how the HSPs are determined for polymers. In this approach, the luminophore is tested in several solvents, and judgements are made as to whether the solvent is “good” or “bad”. This will inevitably result in an “interaction radius” for the luminophore. However, this interaction radius will be completely dependent on the criteria for labelling a solvent “good” or “bad”. We believe that the difference between polymers and luminophores, when it comes to the interpretation of solubility in HSP space, is that it is the distance between the luminophore and solvent HSPs, rather than whether the solvent lies within an interaction radius, which is important. The hypothesis is that the smaller the distance between the platinum-octaethylporphyrin (PtOEP) or coumarin 545T (C545T) HSP values and a given solvent, the better the solubility of the luminophore in that particular solvent. This is different from polymers, where it is simply a question of whether the solvent is within the interaction radius, $R_{0}$. Where the $R_{0}$ for a polymer is something which is fundamental to the polymer itself, the interaction radius of a luminophore instead depends on the criteria used for determining whether the solvent is good or bad, and will therefore act as a kind of “minimum” solubility.

### 1.3. Resonance Energy Transfer and Light Harvesting

**Figure 2:**
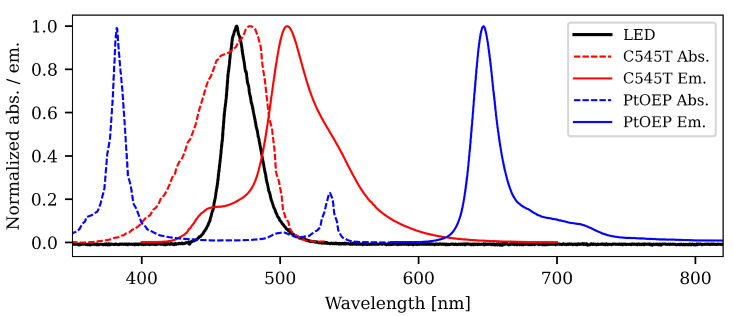
Normalized absorption (dashed lines) and emission (solid lines) spectra of PtOEP (blue) and coumarin 545T (C545T) (red). The LED spectrum is depicted as the solid black curve.

Resonance Energy Transfer (RET) is a mechanism by which overlap in the emission and absorption spectrum (see Figure 2) of two closely spaced luminophores can result in non-radiative transfer of energy from the “donor” molecule to the “acceptor”. This is also known as “light harvesting”. Even though the principle had been used in other sensor contexts before, it was Mayr et al. who, in 2009, proposed the use of light harvesting to increase the brightness in photoluminescent sensors [24].

PtOEP has a low molecular absorption cross-section around 470 nm, which is the center wavelength for the LED used in our work. Much higher absorption is found in the UV. However, UV light tends to damage the PMMA core of the fiber and is therefore not a good candidate as an excitation source. PtOEP has two higher lying absorption peaks at 500 nm and 535 nm, and with a suitable green LED these could be utilized. Green LEDs, however, tend to be less powerful than their blue counterparts, and a trade-off must in such a case be made. In addition, the broad nature of the LED emission spectrum will result in some red light contaminating the back-propagating light, which cannot be removed with a filter, as it will overlap with the PtOEP emission. This effect will be much stronger with a green LED, and it is therefore easier to remove the excitation light from blue LED with a suitable filter than it is with a green.

When coumarin 545T (C545T) is added to the mixture, in suitable quantity, the situation changes drastically. C545T has a very good absorption in the blue region, and its emission spectrum is broad and centered in the green. The emission spectrum therefore overlaps with the absorption peaks for PtOEP at 535 nm. If the PtOEP and C545T molecules are close together, RET [25] may occur and energy is transferred from C545T to PtOEP, without emission from C545T. This will effectively increase the rate of excitation of the PtOEP molecules and thereby result in stronger photoluminescence emission. The emission spectrum of C545T also extends into the red region, and so it could add too the trouble of distinguishing the emission from PtOEP from all the rest. However, it is possible to choose concentrations carefully, such that the emission from C545T is completely quenched.

### 1.4. Phase Fluorometry

Phase-fluorometry uses the effect quenching has on the lifetime of a luminophore, τ, and is a good alternative to intensity-based measurements of quenching, since it is virtually insensitive to losses in the optical fiber, photobleaching of the photoluminescent compound, or drift in the excitation source. The time-constant for the photoluminescence decay, when subject to quenching, can be written in the form of the well-known Stern–Volmer equation

(3) $$\frac{\tau_{0}}{\tau }=1+K_{SV}\left[Q\right]$$

where $\tau_{0}$ is the time-constant for the unquenched system and τ for the quenched system. $K_{SV}$ is the Stern–Volmer coefficient and [Q] is the concentration of the quencher. The coefficient $K_{SV}$ depends on both environmental factors and the quencher. With increasing oxygen concentration, the lifetime of the luminophore decreases.

**Figure 3:**
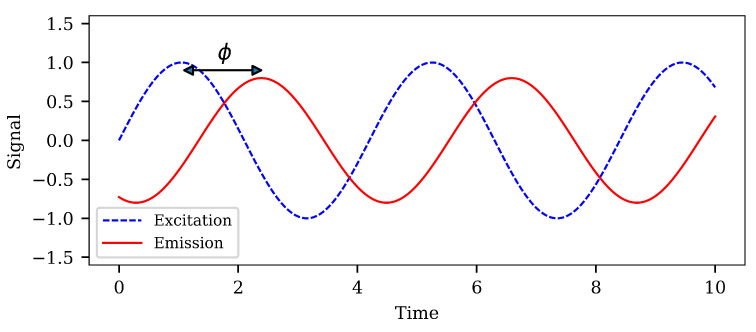
The basics of phase-fluorometry. The excitation signal (blue) is modulated by a frequency $\omega_{0}$. The photoluminescent response (red) has its intensity modulated with the same frequency, but as delayed by an amount ϕ.

When a sample is illuminated by a modulated excitation source with frequency $\omega_{0}$, the emission will also have a modulated intensity with the same frequency. However, depending on the lifetime of the luminophore (and thereby also the oxygen concentration), the emission will be delayed and a phase difference develops (see Figure 3) given by:

(4) $$\phi =\tan^{-1}\omega_{0}\tau$$

## 2. Materials and Methods

The work presented here is divided in three parts. First, a solubility analysis has been performed on the photoluminescent compounds PtOEP and C545T. Next, POF sensors with a layer thicknesses of approximately 45 μm were fabricated with three different sensing-gel mixtures, in order to show the effect of optimizing solubility. Finally, another POF sensor with a thinner layer of about 15 μm was fabricated, to show the capability of producing a fast response-time.

### 2.1. Materials and Equipment

#### 2.1.1. Optical Fiber

The POF was a 1 mm PMMA core fiber with fluorinated polymer cladding (ESKA CK-40) acquired from Edmund Optics. The fibers were jacketed resulting in a 2.2 mm outer diameter.

#### 2.1.2. Polymer

PMMA for the solubility test was acquired from GEHR (www.gehr.de). The solubility testing is described in a previously published work [20].

#### 2.1.3. Solvents

For the solubility tests, the following solvents (>95%) were used: Acetone, 2-propanol, ethanol, trichloroethylene (TCE), dibromomethane (DBM), methyl ethyl ketone (MEK), hexane, chloroform, toluene, dimethyl sulfoxide (DMSO), trans-decahydronapthalene, tetrachloroethylene, cyclohexane, caprolactone, benzyl alcohol, cyclohexanol and 1- bromonapthalene. Acetone and TCE were used in preparing the solutions of polymer, solvent and luminophore. All chemicals were acquired from Sigma Aldrich.

#### 2.1.4. Luminophores

The luminophores used were the phosphorescent PtOEP (>95% acquired from Sigma Aldrich) and fluorescent C545T (>98% acquired from TCI).

#### 2.1.5. Optical Setup

**Figure 4:**
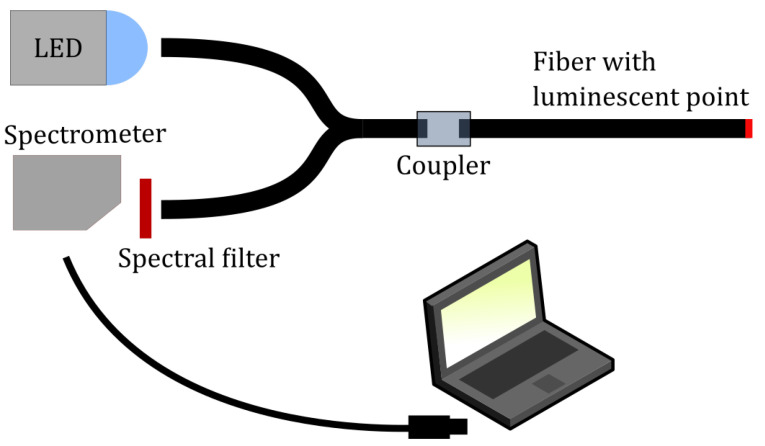
Optical setup for the measurement of photoluminescent intensity from the fabricated sensors. The blue LED is used to excite the luminophores at the distal end of the optical fiber sensor. Emitted photoluminescence propagates back through the system and is directed, through the splitter, to the spectrometer.

Photoluminescence spectra were recorded using an Ocean Optics HR-2000 spectrometer. A simple color-filter from LEE Filters, was used to remove the excitation wavelengths from the back-propagating light. A 1 mm POF splitter was acquired from Fiber-Fin and a blue LED with center-wavelength at 470 nm was used for excitation. The setup is depicted in Figure 4.

#### 2.1.6. Manufacturing Setup

**Figure 5:**
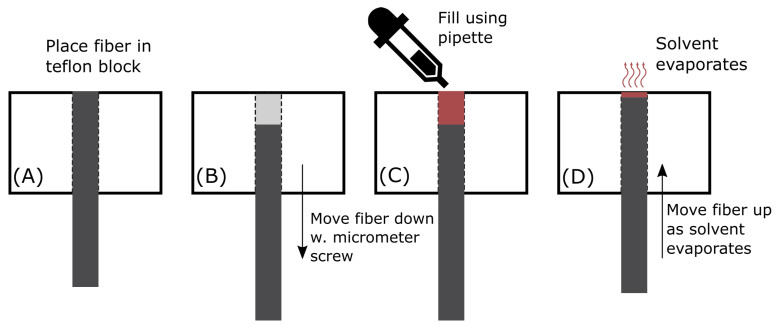
Thin film application principle. In (**A**) the POF is placed in a drilled hole in a Teflon block. A micrometer screw allows for the adjustment of the height of the optical fiber. This is shown in (**B**). In (**C**) a pipette is used to fill the free volume above the fiber and in (**D**) the fiber is slowly moved up, as the solvent evaporates.

A custom-made device was used for applying the sensing layer on the end of the POF. The fiber is mounted in a Teflon block with a 2.2 mm hole drilled through. A micrometer screw allowed for adjustment of the free volume above the fiber end surface. Figure 5 explains the principle behind the device.

### 2.2. Solubility Testing

Solubility testing was carried out by putting a small amount of polymer (approx. 200 mg) or a small amount of luminophore (the tip of a small weighing spatula) into a solvent (15 mL in the case of polymers and 5 mL in the case of luminophores). The exact amount of polymer or luminophore is not important, since the HSPs rely on a qualitative judgement by the researcher on whether a substance is dissolved (or dispersed) or not. What is important is to be fairly consistent with those amounts and judgements.

For polymers, magnetic stirring was used to speed up the process, and after a day or two it was determined whether the solvent dissolved the polymer. If need be, it was possible to wait even longer. The kinetics are not of primary importance, only whether the polymer was dissolved. For more information see the Hansen Solubility Parameters Handbook [18].

For luminophores, the mixture of compound and solvent was first shaken well. Some solvents immediately resulted in a colored mixture. Depending on the solvent the color may be stronger or weaker. However, we let the mixture sit for one day, and then performed a visual inspection of the mixture and look for precipitation. If a precipitate was identified, the solvent was labelled as a “bad” solvent. By using an eye-magnifying glass we also inspect the mixture to see if a suspension was formed. If clumps are visible, the solvent was also marked as “bad”. Using these two judgements, the solvents were labelled as either “good” or “bad”.

The kinetics of dissolution for luminophores and polymers are different. In polymers, the solvent diffuse into the bulk, and plasticize the polymer and allow chains to disentangle from each other. The time for this process depends on a lot of factors, particularly the molecular weight distribution of the polymer and the size of the solvent molecules. It is important to allow polymers the time they need to dissolve, and this is usually one or two days. However, it is possible that even longer times will be required. For luminophores and other particles, dissolution is a surface phenomenon, and so diffusion into the agglomerate is not of importance. This also speeds up the process, and so the timescale will be much shorter than for polymers. It is also worth noting that the surface area of the luminophores (in powder form) is much larger than the surface area of a polymer piece used in our experiments, which is favorable to the kinetics.

For both polymers and luminophores, the software program “Hansen Solubility Parameters in Practice” (HSPiP v. 5.3.02) was used to find the best fit for the cohesive energy densities ($\delta_{D}$, $\delta_{P}$ and $\delta_{H}$), as well as the interaction distances ($R_{0}$). We have previously reported HSPs for the polymers PMMA, Zeonex and TOPAS [20], and will use the values found for PMMA (from Gehr) in this work.

### 2.3. Sensor Fabrication

The sensing gel was created in two steps. Firstly, stock solutions of solvent and luminophore were made in order to precisely control the amount of luminophore added to the sensing mixture. The actual sensing mixture was created by putting polymer and an amount of luminophore stock solution into a small vial. In order to control the viscosity of the mixture, clean solvent was added to make the solution a 15% volume mix of polymer—that is, the polymer accounts for 15% of the total solution volume. We have found that this resulted in a viscosity which was easy to work with. The mixtures made for this work are summarized in Table 1. It is important to note that the concentrations are the concentrations inside the polymer matrix, when the solvent has evaporated.

Next up, the POFs were prepared by cutting them into approximately 20 cm long pieces. Both ends were polished with first a 1200 grit sand paper and then 4000 grit. The POFs were then inserted into the custom-made device for applying the thin film onto the flat tip of the fiber. Depending on the thickness required, the fiber end was lowered to a specific depth. The space above the fiber end-surface was filled with sensing gel by using a suitable pipette. As the solvent evaporated, the fiber was moved up in order to “compress” the thicker and thicker mixture. Depending on the thickness required the fabrication took anywhere from 2 to 10 min. After fabrication the fiber was removed from the fabrication device and dried for at least 16 hours. The day after fabrication, the fibers were each inserted into the setup depicted in Figure 4 and their photoluminescent response was measured.

**Figure 6:**
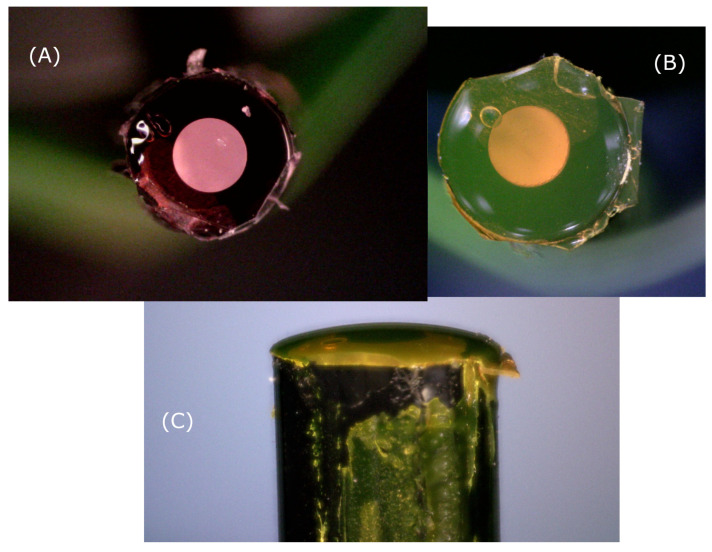
Fiber sensors manufactured by the process of applying a thin film consisting of a PMMA matrix, containing either PtOEP or PtOEP+C545T, at the distal end surface. In (**A**) the fiber is with only PtOEP, while (**B**,**C**) are with a mixture of C545T and PtOEP. The fibers, with jacket, are 2.2 mm in diameter, with a core and cladding of approximately 1 mm. After 16 hours of drying the film thicknesses are on the order of 45 μm.

Initially, three different batches of fibers were created and examples can be seen in Figure 6. The first batch was created with Mix-1 (pure acetone solvent). Acetone is capable of dissolving PMMA, but is less good for PtOEP, although it is possible to create functioning sensors. The second batch was fabricated with Mix-2 (Acetone + TCE mixture as solvent). This solvent mixture is selected since it exhibits good solubility (dissolution) properties for both PMMA and PtOEP—as will be shown in Section 3. The third batch of fibers was created with Mix-3, in which, in addition to PtOEP, C545T is added in order to act as a “light harvester” and thereby enhance the brightness of the light emitted from the PtOEP. As will be explained in Section 3, C545T was easily dispersible in the same solvent mixture used for Mix-2 and Mix-3.

**Figure 7:**
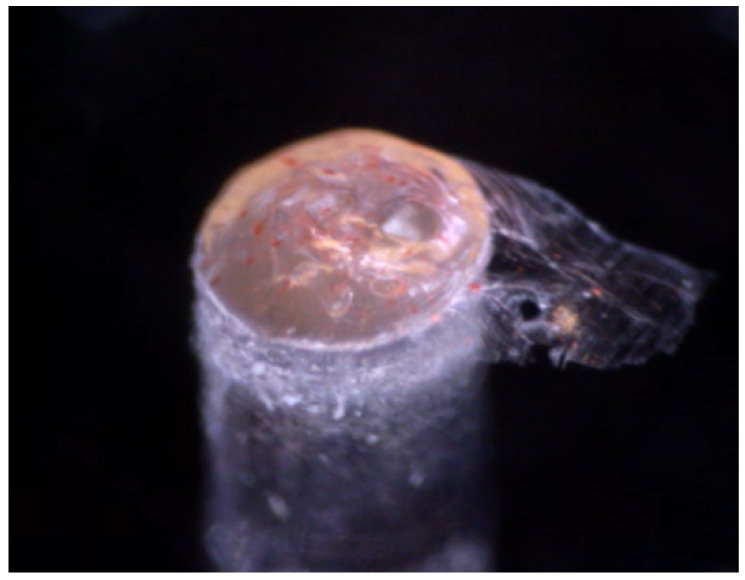
A fiber sensor which has been manufactured with a “bad” solvent. Clumps are visible due to a poor dispersion of the photoluminescent compound (PtOEP in this case). In this particular sensor, the solvent used was acetone.

Using acetone, agglomerates of PtOEP were visible in the polymer matrix. An example of a fiber with clumps formed can be seen in Figure 7.

### 2.4. Oxygen Measurements

**Figure 8:**
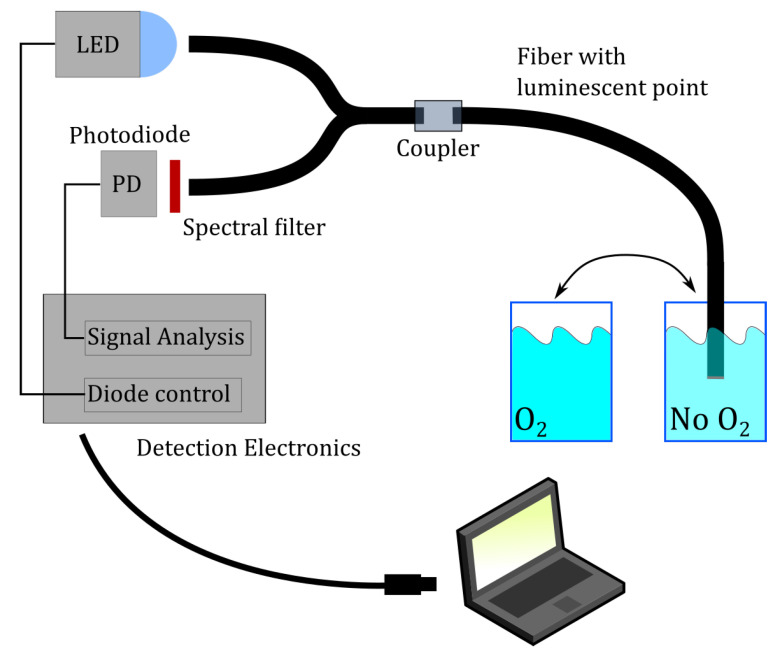
The setup used for oxygen step response measurements. The POF sensor is moved between low-$O_{2}$ and high-$O_{2}$ concentrations and allowed to equilibrate afterwards. The LED is modulated by the electronics system, which can be controlled from a computer. The signal analysis is implemented as a lock-in amplifier (LIA) and extracts an amplitude and phase from the photoluminescence signal. The phase is measured in comparison with the signal driving the LED.

For the oxygen measurements, a special fiber with a thin layer (15 μm) was fabricated with Mix-3. Using Mix-3 allows for the possibility of having a very thin layer, but still a strong signal. Having a thin layer is important in cases where a sensor of fast response time is needed, since the oxygen quickly diffuses into a thin sensing matrix. The measurements were performed in a temperature controlled chamber set to 20 ${}^{\circ }C$. Two volumes of water were placed in the chamber and allowed to equilibrate with the temperature. One of them was then bubbled with nitrogen for at least 1 h to remove dissolved oxygen [26]. The saturation in the low-oxygen water was 0–2% and at the atmospheric water it was 98–100% (measured with an InSitu RDO PRO-X oxygen sensor). The setup can be seen in Figure 8.

The return signal was converted to an electronic signal by way of a photodiode (PD). The signal is recorded and interpreted electronically by a lock-in amplifier (LIA) algorithm, which extracted the amplitude and phase. The phase was measured relative to the excitation signal controlling the blue LED. It is important to note that the system itself will have a certain phase-response, and so it is the differences in phase we are interested in as the oxygen content changes. In practice, the initial measurement performed at low oxygen levels was used as the “baseline”, and this was then subtracted from the phase measured through the rest of the experiment. The modulation frequency of the blue LED is 4000 Hz.

## 3. Results

### 3.1. Solubility

**Figure 9:**
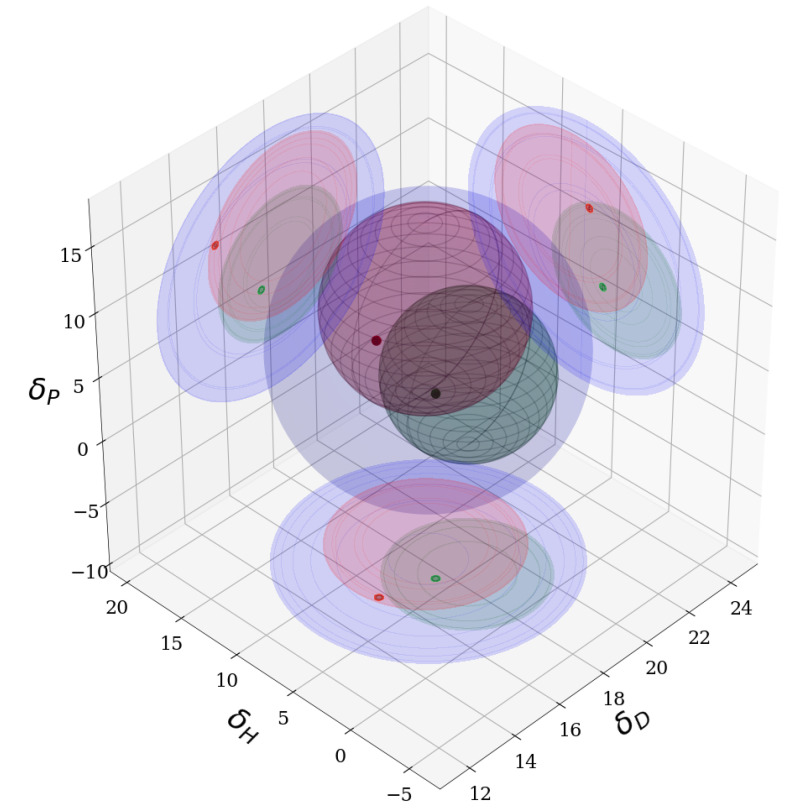
Hansen Solubility Parameter (HSP) spheres for PtOEP (smallest, green), PMMA (middle size, red) and C545T (largest, blue). The 1:3 acetone/trichloroethylene mixture is depicted by a small green dot sitting inside all the spheres simultaneously. Acetone (the small red dot), is outside the solubility sphere for PtOEP. It is also clear that C545T is easily dispersible in any solvents capable of dissolving both PtOEP and PMMA.

Using the approach specified in Section 2.2, HSPs were determined for both C545T and PtOEP. The HSPs for PMMA were taken from earlier work. The results showed clearly that C545T was readily dissolved in many solvents. In fact, all solvents and mixtures capable of dissolving PMMA and PtOEP should also be capable of dissolving C545T. The overlap between PMMA and PtOEP was not total, and so, there exists an intersection between the spheres in which solvents and mixtures must lie, in order for dissolution of PMMA and dispersion of PtOEP to be possible. There are many possible mixtures and solvents which lie within this intersection, and we chose the 1:3 acetone/TCE mixture. The spheres and the positions of both pure acetone and the solvent mixture with TCE can be seen in Figure 9. The HSPs for the luminophores and the solvents can be found in Table 2.

### 3.2. Intensity Measurements

**Figure 10:**
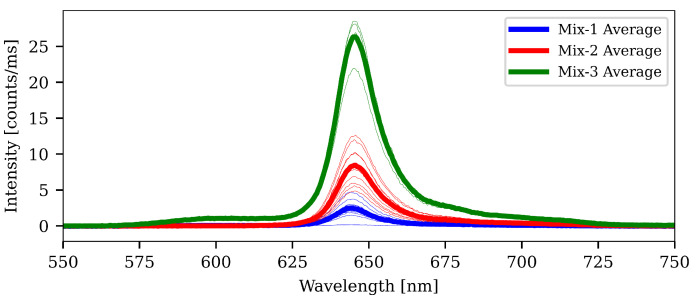
Intensities measured from the different sensors manufactured. All sensors had a concentration of PtOEP of 4.16 mM. The ones made with Mix-1 were the worst of the lot. This is due to the fact that the solvent (acetone) does not provide a good solubility of PtOEP. The result is that PtOEP aggregates into clumps and self-quenching occur. Mix-2 sensors were based on a better solvent (Acetone + TCE), and the intensity is much improved. In that particular solvent, PtOEP can be loaded quite heavily (up to 11 mM according to our measurements). In Mix-3, the added C545T acts as a “light-harvester” and effectively increases the absorption cross-section of the PtOEP.

The results of the intensity measurements are shown in Figure 10. The sensors made with the pure acetone mixture (Mix-1) had the worst performance in terms of photoluminescent emission, and this was expected as PtOEP did not have a good solubility in acetone. The PtOEP had a tendency to aggregate and clump together inside the sensing matrix. The result is that the clumps of PtOEP have a very high local concentration leading to self-quenching.

The acetone and TCE mixture with PtOEP (Mix-2) showed a much better performance—about 3–4 times increase in signal. In the acetone/TCE solvent mixture PtOEP has a much better solubility and the concentration used (4.16 mM) is well dispersed. In such a case no self-quenching occurs. What we have attempted to show here, is that by consciously selecting a solvent mixture based on HSPs, we were able to find a mixture which resulted in complete dispersion of the luminophore for the concentration used. This result is easily extended to other polymers and luminophores.

The next step was to add the light-harvesting compound C545T. The acetone and TCE solvent mix also showed a good C545T solubility, and so C545T was also well-dispersed. The C545T worked as expected by increasing the intensity of the sensor by 2–3 times relative to the result for Mix-2. The effect is attributed to resonance energy transfer occurring between C545T and PtOEP (see Section 1.3). For other luminophores and light-harvesting donors, it may be necessary to compromise and find a “perfect spot” in HSP space for them.

### 3.3. Self-Quenching Concentration

**Figure 11:**
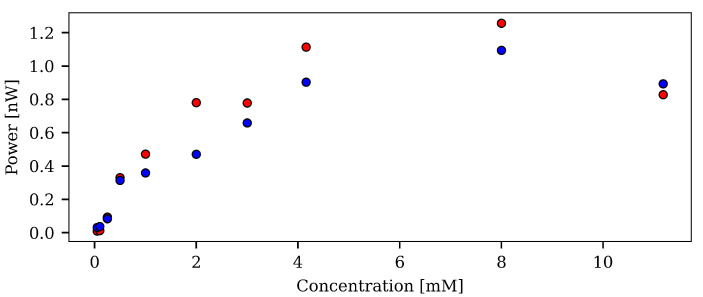
The effect on higher concentrations. Fiber sensors have been manufactured with increasing concentrations of PtOEP using the 1:3 acetone/TCE solvent mixture, ensuring a good dispersion of the compounds. With increasing concentration the effect is, at first, the increase of photoluminescence signal. However, when the concentration rises above 8 mM, the signal drops. It therefore seems that the concentration should stay below this threshold, to ensure that no self-quenching occurs.

High concentrations of photoluminescent compounds will tend to self-quench, even though the fluorophores are well dispersed. In order to test this and ensure that the sensors made with the acetone/TCE solvent mixtures are not in a concentration for which self-quenching occurs, an experiment was performed to evaluate the degree of self-quenching for different concentrations. The results can be seen in Figure 11, and shows that self-quenching began to have a strong effect above 8 mM. The sensors in this study were manufactured with a concentration of 4.16 mM, and we were therefore well below the threshold for self-quenching.

### 3.4. Oxygen Measurements

**Figure 12:**
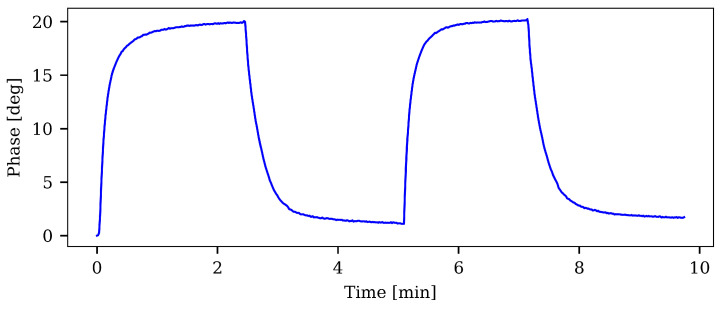
The results of the step-response measurements. The POF sensor underwent two cycles in which it was moved from low oxygen of 0–2% (low phase) to high oxygen of 98–100% (high phase). The $t_{95}$ response-time averaged at 48 s when going from low-oxygen to high-oxygen, and 57 s for the high-to-low transition.

A POF sensor with a thin layer (~15 μm), was manufactured (using Mix-3) and tested to verify that indeed it could measure oxygen. The fiber was placed in the setup depicted in Figure 8 and initially inserted into the de-oxygenated water (0–2%). It was allowed to equilibrate and the phase-response was followed until it no longer changed significantly. The fiber was then moved from the de-oxygenated water to the oxygen saturated water at ambient atmospheric pressure (98–100%). When the phase did not change appreciably it was moved back into the de-oxygenated water and once again allowed to equilibrate. This process was repeated once more. The results can be seen in Figure 12. The response times (from 0–95% of the final value) were 48 s going from low to high and 57 s going from high to low.

## 4. Discussion

Our primary aim with the presented work has been to show the method of using HSPs to quantitatively select solvents for a mixture, in which the luminophores to be used have a good solubility/dispersibility. We have shown that acetone (a bad solvent for PtOEP), results in a lower photoluminescent yield than using a better solvent (the mixture of acetone and TCE), but with the same concentration. This is likely due to the tendency of PtOEP to aggregate and form non-phosphorescent complexes when in pure acetone.

As previously stated, several authors have gone down similar tracks before, and used HSP for the solubility/dispersion of particles. Our hypothesis is that the distance a solvent or solvent mixture has with the luminophore in HSP space, is indicative of the absolute solubility/dispersibility of the luminophore. More work would need to be done to determine how well this fits with reality. A similar study to ours was done by Lee et al. in 2013 [27], in which the authors looked at the solubility matching between a phosphorescent compound and a polymer matrix. They find that matching polymer and phosphorescent compound solubility is important in order to achieve a well-mixed embedding.

In our case, the addition of C545T was straight-forward, since C545T was easily dispersed in many solvents. In fact, as is evident by the results in Figure 9, C545T will be dissolvable in any solvent or solvent-mix, which works for either PtOEP or PMMA. However, this may not always be the case. It is possible to use different donors and acceptors in other applications, and they may not be so readily mixed together in a single solvent. The approach we have outlined here, gives researchers a tool they can use, in order to investigate whether a compromise between two hard to dissolve luminophores exist.

In our experiments, we also used PMMA as the polymer matrix. For oxygen sensing, there are other polymers, which have higher permeability to oxygen, which will therefore give rise to even shorter response times as well as larger phase shifts. This, however, is not always what is wanted. Large phase-shifts also mean that the oxygen will dampen the luminescence much more, and the system will therefore receive a smaller signal. However, if a different polymer is to be used, it is easy to find a new solvent or mixture, which would work well. One needs only to perform a solubility analysis of the polymer to find the HSPs, and then analyse the intersection between the luminophore solubility spheres and that of the new polymer. Solvent mixtures can then be formulated by clever selection of ratios.

Finally, a sensor could be envisioned in which several different luminophores are used as indicators of different parameters (temperature, oxygen, pH). In those instances, the simultaneous solubility of the compounds may indeed be difficult to achieve. However, using HSPs one can once again attempt to find an overlap between the three solubility spheres and formulate a solvent which satisfies the needs.

## 5. Conclusions

We have presented an all-polymer fiber optical sensor for dissolved oxygen with the sensing matrix fabricated by quantitatively finding a good solvent mixture capable of dispersing a high amount of the luminophore (PtOEP) as well as dissolving the polymer (PMMA) leading to a strong molecular entanglement adhesion to the PMMA fiber end-surface. We presented the approach of determining HSPs for luminophores and used this approach for both PtOEP and the brightness enhancer C545T. Using C545T in conjunction with PtOEP, it is possible to significantly increase the brightness of the oxygen sensor. This allows for the possibility of having a very thin layer at the end of the fiber, with a resulting fast response-time. In our tests we succeeded in making a sensor with a $t_{95}$ response-time of less than 1 min for both up and down step oxygen concentration measurements.

Our sensors are the first fiber-optical oxygen sensors utilizing light harvesting molecules, and using our approach for optimal solvent selection, the fabricated sensors showed a three-fold increase in signal intensity and this can be improved further with different concentrations of PtOEP and C545T.

The approach of using HSPs to determine the dispersibility of a luminophore in solvents, is a practical and useful tool for researchers working with polymeric matrices and luminophores. Using this approach, it is possible to find solvents capable of dispersing high-concentrations of one or more luminophores. However, more work remains, and especially the question whether a direct relationship between the solvent-luminophore distance in HSP space and absolute solubility exists.

## Figures and Tables

**Table 1:** Mixtures used for sensor fabrication.

Mix	Solvent	PtOEP	C545T
Mix-1	Acetone	4.16 mM	
Mix-2	Acetone + TCE (1:3) (*v/v*)	4.16 mM
Mix-3	Acetone + TCE (1:3) (*v/v*)	4.16 mM	12.23 mM

**Table 2:** HSPs for luminophores and solvent mixture.

	$\delta_{D}$	$\delta_{P}$	$\delta_{H}$	$R_{0}$
PtOEP	18.28	5.98	4.61	5.5
C545T	17.94	6.77	7.38	10.1
Acetone/TCE (1:3)	17.4	4.9	5.7	
Acetone (pure)	15.5	10.4	7	
The units of the HSPs and $R_{0}$ are [MPa${}^{1/2}$].				
